# Protonated and Cationic Helium Clusters

**DOI:** 10.3390/molecules25051066

**Published:** 2020-02-27

**Authors:** Linnea Lundberg, Peter Bartl, Christian Leidlmair, Paul Scheier, Michael Gatchell

**Affiliations:** 1 Institut für Ionenphysik und Angewandte Physik, Universität Innsbruck, Technikerstr. 25, A-6020 Innsbruck, Austria; 2 Department of Physics, Stockholm University, 106 91 Stockholm, Sweden

**Keywords:** helium clusters, protonation, helium droplets, noble gases, mass spectrometry

## Abstract

Protonated rare gas clusters have previously been shown to display markably different structures compared to their pure, cationic counterparts. Here, we have performed high-resolution mass spectrometry measurements of protonated and pristine clusters of He containing up to 50 atoms. We identify notable differences between the magic numbers present in the two types of clusters, but in contrast to heavier rare gas clusters, neither the protonated nor pure clusters exhibit signs of icosahedral symmetries. These findings are discussed in light of results from heavier rare gases and previous theoretical work on protonated helium.

## 1. Introduction

One of the first things a chemistry student learns when encountering the periodic table is that elements in the last column, group 18, or the noble gases, are unreactive and do not form chemical bonds. Unlike the other elements, that in most cases are only ever found in molecular compounds, be they homonuclear or heteronuclear, we learn that the noble gas elements exist as solitary atoms. This is, as it turns out, only part of the truth. Strongly electronegative ligands, such as O and F atoms, have been found to be able to form neutral, covalently bound structures with Ar, Kr, and Xe, with the latter in particular displaying a rich chemical nature, but neutral compounds containing bound He and Ne have generally not been achieved [1,2,3]. Exotic exceptions to this are compounds formed under extreme pressures [4] or by trapping noble gases in molecular cages [3,5,6]. However, molecular *ions* containing covalently bound rare gases, including He and Ne, can be readily formed and have been studied since the discovery of the HeH${}^{+}$ ion nearly a century ago [7].

Ensembles of neutral rare gas atoms interact through weak van der Waals forces and can condense into clusters at low temperatures. The progression of geometries with increasing cluster sizes generally starts out by the filling of atoms into icosahedral (sub-)shells, with characteristic shell closures at sizes of 13, 55, and 147 monomers [8,9]. At cluster sizes greater than a few dozen atoms, non-icosahedral structures displaying, e.g., decahedral and octahedral symmetries may also form the energy minima at certain unique sizes [9,10,11,12]. For ionic rare gas clusters however, the cluster geometries are more complex as the charge significantly influences the interactions between the atoms. Starting in the early 1980s, a large body of work began to emerge as cationic clusters of rare gas atoms were produced by the expansion of cooled gases and studied by means of mass spectrometry. One of the earliest such studies focused on xenon clusters [13]. That study showed an excellent agreement between the experimental results and a simple sphere packing model and confirmed that the clusters indeed have icosahedral symmetries [13]. Later studies largely confirmed these findings for other rare gas species [14,15,16,17,18,19,20,21], but discrepancies emerged between different measurements that sparked debate. Most notable were the differences observed in competing studies of Ar${}_{n}^{+}$ clusters, where reported abundance anomalies deviated without explanation [15,16,17,18,21,22,23,24]. The disagreement remained unexplained for nearly three decades, during which the consensus was that different cluster geometries were obtained depending on whether they were born neutral (and then ionized before detection), or grew around an ionic nucleus [25]. These discrepancies were only recently explained in a study on protonated rare gas clusters that showed the presence of protonated clusters in some measurements could be the underlying cause [26]. In that work, high-resolution mass spectrometry was used to compare the differences between pure, cationic argon clusters and equivalent systems also containing a single H atom. The protonated cluster series showed a much better match with sphere packing models and measurements from Xe${}_{n}^{+}$ clusters [13] than the pure cationic clusters did [26]. Furthermore it was shown that there were significant differences in the magic numbers between the protonated and cationic systems. In the pure clusters, the charge is carried by a linear Ar${}_{n}^{+}$ ion, with 2≤n≤4. Compared to the interactions between neutral atoms, this ion is significantly contracted. The different bond lengths in this ion compared to the interaction distances between the surrounding neutral atoms break the symmetry of the clusters and distorts their geometries, preventing the formation of particularly stable icosahedral structures [26]. In the protonated clusters, the charge center consists of an (Ar-H-Ar)${}^{+}$ ion, where the separation between the two rare gas atoms is close to the distance between two neutral atoms. This preserves the overall symmetry of the systems and explains why the protonated clusters better match models (which are often based on neutral clusters) [26]. A followup study on Ne and Kr clusters showed that the structural effect of protonation is strongest for lighter rare gases and decreases in strength for the heavier species [27]. It is therefore not expected to play a significant role for Xe clusters. The discrepancies between past studies were thus understood as being the result of unresolved protonated cluster series, most likely formed by breakup of residual water molecules that are often present in experiments.

Protonated He atoms and clusters are attractive systems from a theoretical standpoint, due to the relatively small number of electrons and the nuclear quantum effects present with light atoms, and have been the focus of numerous computational studies [28,29,30,31,32,33,34,35]. Experimentally, small protonated He clusters have, for example, been produced from He nanodroplets doped with molecular hydrogen for mass spectrometry and spectroscopic studies [35,36,37], and in low temperature drift tubes filled with He gas [38]. Consisting of the two most abundant elements in the universe, HeH${}^{+}$ ions and complexes containing this ion as a chromophore are expected to be plentiful in astrophysical plasmas [39,40,41], but was only very recently discovered in spectra obtained by the SOFIA airborne observatory [42].

In this paper we present measurements of cationic and protonated clusters of He produced from pure and doped helium nanodroplets. We identify both types of clusters containing up to 50 He atoms and compare the differences in abundance anomalies, which reveal information regarding differences in their structures. These results are discussed in light of recent results on other protonated rare gas clusters [26,27].

## 2. Results and Discussion

**Figure 1:**
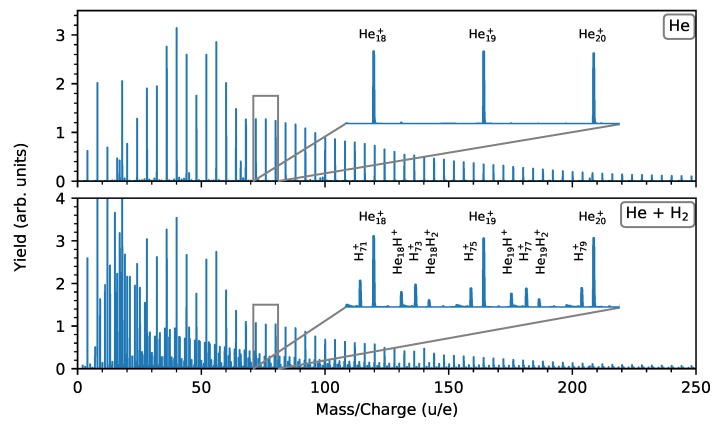
Mass spectra from the ionization of pristine He droplets (top panel) and droplets doped with molecular hydrogen (bottom panel). In the latter, cluster series from different mixing of He and H are present, most prominently from He${}_{n}$H${}^{+}$, H${}_{x}^{+}$, and He${}_{n}$H${}_{2}^{+}$ clusters. Small peaks from residual gas ions are also visible in both spectra and their presence is corrected for in the data analysis.

Mass spectra obtained from the ionization of He droplets by electron impact are shown in Figure 1. In the top panel, pristine droplets were used. When helium droplets are ionized by electrons with energies above the ionization threshold of He atoms (24.6 eV), positive charges can accumulate within them [43]. If the repulsive forces of the accumulated charges overcome the cohesive forces of the droplets, excess charges will be emitted in the form of small cationic fragments (with a total mass of less than a few percent of that of the parent droplet) [43]. The mass spectrum in the upper panel shows a distribution of such fragments. The clusters follow a broad size distribution with a maximum between 10 and 20 He atoms and with a tail that extends to much larger sizes. Within the overarching distribution, a number of abundance anomalies are visible. Most notable is the minima for He${}_{12}^{+}$ clusters.

In the bottom panel of Figure 1, a mass spectrum from droplets doped with H${}_{2}$ prior to ionization is shown. The dominant cluster series in this data is once again the set of He${}_{n}^{+}$ peaks. These show the same features that are present in the measurement of pristine droplets (top panel). However, the pristine spectrum is still used for the analysis of pure He${}_{n}^{+}$ clusters in order to avoid overlap with other cluster series (mostly H${}_{x}^{+}$ clusters). Other prominent features in this spectrum are peaks from He${}_{n}$H${}^{+}$, H${}_{x}^{+}$, and He${}_{n}$H${}_{2}^{+}$ clusters, which follow their own broad size distributions (resulting from the pickup conditions in the experiment). Additionally, features arising from higher order mixtures of He and H are present as weaker peaks in the spectrum. Species containing odd numbers of H atoms are formed by the breakup of an H${}_{2}$ molecule upon ionization and are here referred to as protonated clusters. The insets in the two panels of Figure 1 show the same zoomed-in mass region of both data sets. The difference between them is clear, with the bottom panel displaying additional peaks between the pure He${}_{n}^{+}$ masses. The high-resolution of these measurements (m/Δm∼6000) allows us to separate the different features in the mass range shown here, but due to the different mass defects of He and H the peaks drift relative to each other and start to overlap at higher masses. For this reason we have limited our analysis in the rest of this paper to clusters containing 50 He atoms or less.

**Figure 2:**
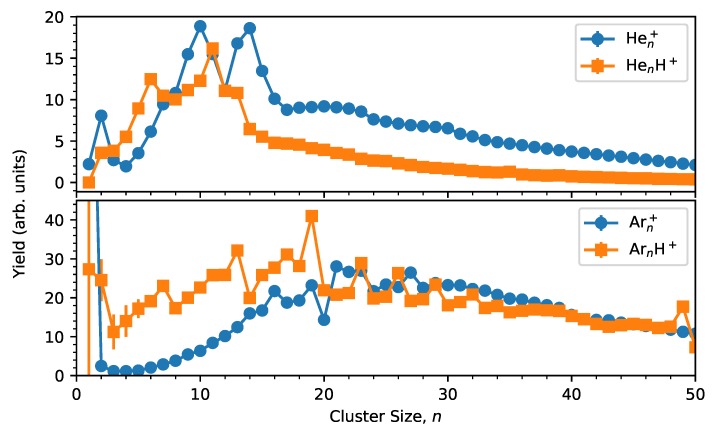
Extracted distributions from measurements of He${}_{n}^{+}$ and He${}_{n}$H${}^{+}$ clusters (top panel). In the bottom panel, data for argon clusters from ref. [26] are shown for comparison. The statistical uncertainties are, for almost all of the points, smaller than the marker size.

Integrated ion yields, extracted using IsotopeFit [44], are shown as a function of cluster size in Figure 2. The upper panel in this figure contains data from the present measurements on He clusters, while the lower panel shows equivalent data for pure and protonated Ar clusters from ref. [26]. The pristine He${}_{n}^{+}$ clusters display a strong dimer peak as the first main feature. The tightly bound He${}_{2}^{+}$ ion is one of the main charge carriers in cationic He droplets [45] and its abundance relative to larger clusters can be used to gauge the size of the parent droplets from which the clusters are formed [46]. Larger cluster sizes with clear abundance anomalies include the magic n=10 and 14, as well as the strong dip at n=12, which can be considered as anti-magic. These features are all well-known from past studies on positively charged He clusters [46].

**Figure 3:**
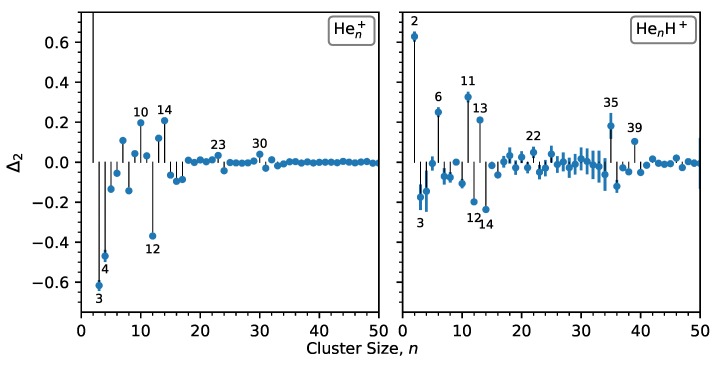
Second differences in ion yields (defined in Equation (1)) for He${}_{n}^{+}$ (left panel) and He${}_{n}$H${}^{+}$ clusters (right panel). Positive values indicate relatively abundant cluster sizes and negative values less abundant sizes.

The protonated cluster series deviates significantly from the pristine series. None of the magic features in the pristine cluster series are present in the protonated series and a new set of magic numbers now appear. The standout features for He${}_{n}$H${}^{+}$ clusters are the magic sizes of n=6 and 11, with other weaker anomalies found at n=2 and 13. In Figure 3 we also show the second difference for each cluster size, which is defined as:

(1) $$\Delta_{2}=\log\left[\frac{I_{n}}{\frac{1}{2}\left(I_{n-1}+I_{n+1}\right)}\right],$$

where $I_{n}$ is the integrated ion yield for cluster size *n* and log is the natural logarithm. This expression determines the intensity of a cluster size relative to its neighbors, and reduces the influence of the underlying statistical size distribution. Positive values correspond to particularly strong features, and conversely, negative values represent locally weak features. From Figure 3 we can identify some weaker abundance anomalies in the mass spectra. For the pristine clusters these are the sizes n=23 and 30, and for the protonated clusters, n=35 and 39 particularly stand out.

Comparing the results from He clusters with those from Ar clusters (bottom panel of Figure 2) we see that helium clusters, regardless of whether they are cationic or protonated, show very little agreement with icosahedral shell closures. The Ar${}_{n}$H${}^{+}$ series shows an almost perfect agreement with such models [13,26], but only one of the magic numbers associated with icosahedral structures, n=13, is found for He${}_{n}$H${}^{+}$. However, other magic sizes that are characteristic for protonated clusters of heavier rare gases, such as n=7 and 19, and which also signify icosahedral structures, are missing for protonated He. Theoretical calculations of He${}_{n}$H${}^{+}$ structures suggest that sizes of n=6 and 13 are particularly stable, in good agreement with our findings, but surprisingly show no enhanced stability for n=11 [35], which is the strongest abundance anomaly in our data. The binding energies determined by the calculations show that atoms in the first solvation layer around the proton (3≤n≤6) each have a binding energy of about 250 meV per atom [35]. The second layer atoms (up to n=13) are bound by approximately 6 meV per He, while subsequent layers are bound even more weakly, with the binding energy eventually approaching that of neutral clusters (≈0.6 meV per atom) [35,47]. The calculations also show that the structures do not display icosahedral symmetry or any other higher order level symmetry for most sizes [35], unlike other protonated rare gas clusters [26,27].

Highly symmetric cluster geometries, like icosahedral structures, are typically achieved for systems where all of the bond lengths in the lattice are equal. Cationic rare gas clusters will to some degree always contain a defect due to the contracted charge center. For Ar clusters [26], and to a lesser degree Ne and Kr clusters [27], protonation largely repairs the broken symmetry imposed by the charge, but this is clearly not the case for He clusters. The calculated (He-H-He)${}^{+}$ length of 1.85 Å [35] is indeed significantly longer than the He${}_{2}^{+}$ bond length of 1.08 Å [48], but still dwarves the typical He-He distance of ≈3.58 Å in neutral droplets [48]. There is also an incompatibility with the He atoms solvating the protonated core of the cluster, with the first solvation layer occupying a distance of about 2.1 Å from the central He atoms [35]. It is thus not surprising that the cationic He clusters, both pure and protonated, do not display distinct magic numbers consistent with packing the atoms into icosahedral solvation layers. A more detailed approach, one that includes quantum effects of the nuclear motion, would likely be needed to fully explain the structural differences between the cationic and protonated He clusters and is left for the future.

## 3. Materials and Methods

Helium nanodroplets (with typical sizes around $10^{6}$ He-atoms) are produced by pre-cooling compressed (25 bar) helium (Messer, 99.9999% purity) to a temperature of 9 K and releasing it through a 5 μm wide nozzle into the experimental apparatus. The helium droplet beam then goes through a 0.8 mm diameter skimmer that is placed 8 mm from the nozzle. The droplets pass through a series of pickup cells where, in one of the experiments, H${}_{2}$ gas is introduced through a gas line and are picked up in collisions with the helium droplets. The doped droplets are then ionized by electron impact using 64 eV kinetic energies (with an energy spread of about 1 eV). This energy is well above the ionization potential of He (24.6 eV) and is chosen to optimize the experimental conditions such as beam overlap (the exact energy has little effect on the resulting cluster distributions). The positively charged clusters are then analyzed and detected using a reflectron time of flight mass spectrometer (Tofwerk AG model HTOF). Additional experimental details can be found in refs. [49,50,51].

In the ionization process, an electron is removed from a He atom in the droplet forming He${}^{+}$. The positive charge will then typically migrate through the droplet from one He atom to another about ten times (on average) via resonant hole-hopping before a He${}_{2}^{+}$ ion is formed [45]. This ion forms the nucleus around which clusters are formed, with neighboring atoms being attracted by the charge. In droplets doped with H${}_{2}$, the difference in ionization energy between helium and hydrogen will lead to the breakup of one of these molecules and the appearance of an H${}^{+}$ ion, which can result in a protonated cluster. This method of producing rare gas clusters has been used in the past to study other types of rare gas clusters [24,26,27,49], giving results in good agreement with other techniques [15,18,20,22,23].

The data is analyzed using the IsotopeFit software [44]. This software fits custom peak profiles to the experimental mass spectrum while considering the molecular species in the sample and the respective isotopic abundances. It extracts the relative abundances of each species, correcting for the isotopic patterns and background noise levels, resulting in the different cluster series distributions.
